# Heart-warming hip preservation

**DOI:** 10.1093/jhps/hnx043

**Published:** 2017-12-06

**Authors:** Richard (Ricky) Villar

**Affiliations:** Editor-in-Chief, Journal of Hip Preservation Surgery

There is something incredibly heart-warming about attending the annual ISHA meeting, this year in Chile’s Santiago. It happens to me each time. I was trying to work out why that might be, being heart-warmed, that is. Was it the coming together of like-minded colleagues—well, friends actually—from around the world, bound by a common interest? Was it the realization that younger blood is now shining through and the field of hip preservation is, at long last, secure? Or, was it the astonishment I felt when I visited a brilliant poster section that had more than 100 papers displayed, each with a message that would improve patient care, each from a unit of considerable professional standing? 

Hip preservation, thanks to the efforts of many, is now here to stay. Impingement surgery, too, has just come through its first RCT and has been shown to be more than effective and certainly better than conservative, non-operative care. Anyone who had the opportunity to hear Professor Damian Griffin present the multicentre work from around the world will agree, I feel sure, that his was an exemplary presentation. Sadly, I had no video camera handy to record it but it was an example to public speakers globally of how to transmit a professional message and yet keep your audience on the edge of their seats. Eat your heart out TED presenters, we have got you beat. Next step, I might suppose, will be watching insurers and Governments work out how they might do down such excellent research. They have been clamouring for an RCT, they now have an RCT—and with others to shortly follow—so let us see if they stand by their word and help rather than hinder hip preservation.

I was a little worried in Santiago that everything I heard was positive. Is hip preservation so fantastic that everything works as expected? If so, that is not real life. I mentioned then, and I mention now, might there not be room for a few papers with negative findings? What does not work is sometimes more important than finding out what does. More to follow about this, I suspect.

The last issue of *JHPS* was, as ever, a mine of new findings. If you have missed it, please read it now. I especially enjoyed Haldane *et al*.’s systematic review [1] of physical examination and imaging for impingement. One can never have enough of such papers as they are so applicable to hip preservation practice. Meanwhile the paper by Johnson *et al*. [2] about the use of ipsilateral femoral head osteochondral transfers for osteochondral defects of the femoral head will be of immense value, I wager, for anyone who sees avascular necrosis in practice.

And as for this issue, Issue 4.4, once again I am spoilt for choice. My choice of one title, maybe two, is not to do down the others as all, in my view, are brilliant. What about the piece on osteopetrosis by Reinhold Ganz *et al*. [3]? Theirs is an almost unique experience, yet osteopetrosis is something that does walk through our clinic doors on occasion. We need guidance on what to do and this paper does precisely that. And I know we are not supposed to publish case reports but sometimes, just sometimes, you feel you must, as their message is quite fascinating. So, have a look at the Clinical Vignette by Nikolaos Davarinos *et al.* [4] on spontaneous ligamentum teres reattachment after surgical dislocation of the hip. I know I am more than biased but, to be frank, I thought the illustrations for this case were remarkable.

So, welcome once again to another issue of *JHPS*, your journal, our journal, the journal for the hip preservation world. Please be sure to read it, digital cover-to-cover will do.

Oh yes, and please do not miss the annual ISHA meeting in 2018, held in Melbourne, Australia. I, for one, have already booked my flight.

My very best wishes to you all.
